# Leaf Length Tracker: a novel approach to analyse leaf elongation close to the thermal limit of growth in the field

**DOI:** 10.1093/jxb/erw003

**Published:** 2016-01-27

**Authors:** Sebastian Nagelmüller, Norbert Kirchgessner, Steven Yates, Maya Hiltpold, Achim Walter

**Affiliations:** ^1^Institute of Agricultural Sciences, Swiss Federal Institute of Technology,Universitätstrasse 2, 8092 Zurich, Switzerland; ^2^Institute of Botany, Department of Environmental Sciences, University of Basel, Schönbeinstrasse 6, 4056 Basel, Switzerland

**Keywords:** Field conditions, leaf elongation, low temperature, marker tracking, monocotyledons, phenotyping, plant growth.

## Abstract

Leaf growth in monocot crops such as wheat and barley largely follows the daily temperature course, particularly under cold but humid springtime field conditions. Knowledge of the temperature response of leaf extension, particularly variations close to the thermal limit of growth, helps define physiological growth constraints and breeding-related genotypic differences among cultivars. Here, we present a novel method, called ‘Leaf Length Tracker’ (LLT), suitable for measuring leaf elongation rates (LERs) of cereals and other grasses with high precision and high temporal resolution under field conditions. The method is based on image sequence analysis, using a marker tracking approach to calculate LERs. We applied the LLT to several varieties of winter wheat (*Triticum aestivum*), summer barley (*Hordeum vulgare*), and ryegrass (*Lolium perenne*), grown in the field and in growth cabinets under controlled conditions. LLT is easy to use and we demonstrate its reliability and precision under changing weather conditions that include temperature, wind, and rain. We found that leaf growth stopped at a base temperature of 0°C for all studied species and we detected significant genotype-specific differences in LER with rising temperature. The data obtained were statistically robust and were reproducible in the tested environments. Using LLT, we were able to detect subtle differences (sub-millimeter) in leaf growth patterns. This method will allow the collection of leaf growth data in a wide range of future field experiments on different graminoid species or varieties under varying environmental or treatment conditions.

## Highlight

A novel weatherproof *in situ* method for high-precision leaf growth measurements in monocot plants was used to detect genotype-specific growth responses even at low temperature.

## Introduction

Provided soils are moist and fertile, leaf growth dynamics in monocot plants are largely determined by temperature, which has been shown in several grass taxa, including wheat (*Triticum aestivum*), barley (*Hordeum vulgare*), maize (*Zea mays*), and wild grasses (*Lolium perenne*) (Bliss, 1956; Watts, 1971; Peacock, 1975a; Peacock, 1975b; Gallagher and Biscoe, 1979; Gallagher *et al.*, 1979; Körner and Woodward, 1987; Sadok *et al.*, 2007). The response time of graminoids to temperature is very short; growth rates react within a few minutes to temperature changes and follow the thermal course of their treatment or environmental conditions unless no other important abiotic factors, such as water availability and nutrient supply, constrain plant growth (Peacock, 1975a; Stoddart *et al.*, 1986; Pollock *et al.*, 1990; Walter *et al.*, 2009). However, the sensitivity of plant growth to temperature varies among species. Cold-adapted winter cereals or arctic alpine grasses maintain leaf growth under very low temperatures, reaching a limiting temperature (so-called base temperature T_b_) close to 0°C (Gallagher and Biscoe, 1979; Gallagher *et al.*, 1979; Körner and Woodward, 1987), at the price of lower maximum rates at warm temperatures. For example, lowland ryegrass (*Lolium perenne*) has a T_b_ close to 4°C (Peacock, 1975b) and maize, which originates from warm climates, has a T_b_ close to 10°C (Reymond *et al.*, 2003).

The physiological reasons for the strong differences bet ween species with respect to low temperature effects on plant growth are complex. In sensitive monocots such as maize, low temperature diminishes cell production and increases the cell cycle time (Rymen *et al.*, 2007), which contributes to a lower leaf elongation rate (LER). Cold- adapted plants maintain cell division until freezing (Körner and Pelaez Menendez-Riedel, 1989); and have not been found to be carbon source limited at 5°C, where they still perform 50–70% of their assimilation provision (Körner 2003). It is likely that metabolic processes involving tissue formation and differentiation are the most temperature sensitive (Körner 2015) and thus limit leaf elongation at low temperatures.

Above T_b_, the temperature response of LER is currently less clear than might be anticipated. For example, in some studies with cold-acclimated winter cereals and ryegrass, the relationship between growth and temperature has been shown to be linear (Gallagher, 1979), whereas an exponential response has been reported (Peacock, 1975a; Peacock, 1975b) in a temperature range between ~4°C and 20°C. Others have described the response curve of growth to temperature as a combination of linear and non-linear functions, with an exponential component dominating just above T_b_, a linear component dominating at intermediate temperatures, and saturation towards high temperatures (Blum, 1986; Voorend *et al.*, 2014).

Increasing our understanding of physiological and environmental factors affecting growth in the field requires *in situ* non-destructive methods. Serial analyses of the same organ is essential, given that the inter-individual variability of organ size strongly limits the resolution when using destructive methods, even when they are based on a high number of replicates (Walter *et al.*, 2009). Therefore, analysis of leaf area as a proxy of dry weight (Briggs *et al.*, 1920) has long been used and has proved to be a valuable tool in contemporary physiological (Walter *et al.*, 2009) and agronomical studies (Furbank and Tester, 2011; Hartmann *et al.*, 2011; Fiorani and Schurr, 2013).

Measurement of leaf elongation is a precise tool with which to study plant growth in grasses. Methods have been developed and carried out since the early 20th century using the classic auxanometer approach (e.g., Bovie, 1912; Bovie, 1915; Koningsberger, 1922; Idle, 1956; Ranson and Parija, 1955). With further improvements, these methods have become essential tools in understanding growth and the response of plants to their environmental conditions. In monocot plants, a variety of mechanical methods measuring linear extensions or LERs have been successfully established using linear variable displacement transducer or rotary resistance transducer techniques (Gallagher *et al.*, 1976; Körner and Woodward, 1987; Ben-Haj-Salah and Tardieu, 1995; Poiré *et al.*, 2010). More recently, optical approaches based on time-lapse imaging of monocot leaves or canopies have been established (Poiré *et al.*, 2010; Hartmann *et al.*, 2011; Matos *et al.*, 2014; Grieder *et al.*, 2015). However, these previous approaches have not yet achieved a combination of high temporal resolution and high throughput in LER analysis in the field. Such a combined analysis is necessary to uncover more details of leaf growth processes, not only at low temperatures, and would represent a major success for plant science and breeding.

In this paper, we present a novel method to measure LER that can be used in the field as well as under controlled conditions. The method is a hybrid between the classic mechanical approach and an imaging-based marker tracking approach described by Mielewczik *et al.*, (2013). It combines the advantages of precise elongation analyses with an automated, cheap, and weatherproof image-based recording unit that monitors considerably higher sample sizes of leaves simultaneously using only one measurement unit (camera), thus allowing the determination of reliable growth rates.

We intended to test whether the set-up and approach can (a) be applied in different experimental settings (field and climate chamber), and (b) provide enough statistical power to differentiate between LER-temperature response curves under the difficult condition of low temperature.

## Material and methods

### Plant material and experiments

Four winter wheat varieties (*Triticum aestivum* L., varieties ‘Combin’, ‘Caphorn’, ‘Cambrena’, and ‘Chaumont’), three distichous spring barley varieties (*Hordeum vulgare* L. f. *distochon*, varieties ‘Ascona’, ‘Eunova’, and ‘Quench’) and six rye-grass varieties (*Lolium perenne*) were grown in the following experimental settings.

**Fig. 1.**
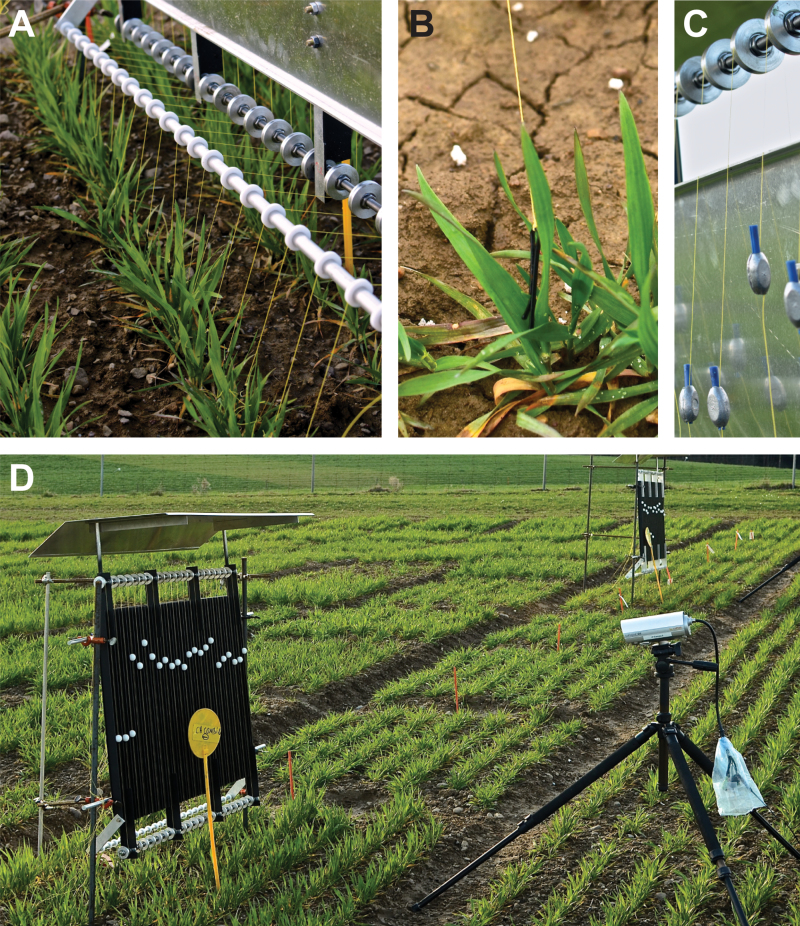
Experimental set-up of the measurement panel in the field. Similar panels without a roof were used in climate cambers. (**A**) Row-wise measurement of 20 wheat leaf replicates. Here, it is visible how threads are passing the first and second reverse rollers. (**B**) Close up view of a leaf tip attached by a hairpin. (**C**) The 20g counterweight and third reverse roller. (**D**) Wheat micro plots and the installed measurement panels with white beads and a near-infrared camera in front. The first three beads on the left were used for reference measurements. The panel was north facing to avoid shading of the investigated plants. The panel roof was installed to prevent confounding effects by raindrops and snow on the beads.

(i) Winter wheat in a field setting: winter wheat was cultivated in spring season of 2014 in plots of 1.5×1.5 m in rows of 17cm distance at the ETH research station for plant sciences Lindau-Eschikon (‘Eschikon’; 47.449°N, 8.682°E, 520 m above sea level; soil type, gleyic cambisol; sowing date, 19th October 2013). The plots were part of a larger experiment described in Grieder *et al.* (2015). Leaf elongation measurements were made with 20 replicates per variety in two consecutive weeks (week 1 & 2) from 25th March 2014 to 7th April 2014 (Fig. 1D).
(ii) Spring barley in a field setting: spring barley was grown in plots of 1×2 m in two contrasting field sites in rows 20cm apart, at Eschikon (sowing date: 7th April 2014) and on a southeast exposed mountainside in Kunkels, Switzerland (46.873°N, 9.409°E, 1180 m above sea level; soil type, calcareous chernozem; sowing date, 10th April 2014). Five weekly measurements were made with seven replicates per variety from 22nd April 2014 to 30th May 2014.
(iii) Spring barley in a climate chamber: during the same period as for the barley above, spring barley was grown in a climate chamber (Conviron, Winnipeg, MB, Canada; sowing date, 4th April 2014) in pots (10×10×20cm, nine plants per pot) filled with a 4:1 mixture of soil (Landerde, Ricoter, Aarberg, Switzerland) and silica sand (0.5–2mm diameter). The illumination cycle was comparable to the barley field conditions with a day/night period of 13/11h reaching a light intensity of 275µmol of photosynthetically active radiation (PAR) m^−2^s^−1^. The average day/night temperature was 10/2°C, and relative humidity was kept at 60±15%. Measurements were made with seven replicates per variety from 17th April 2014 to 20th May 2014.
(iv) Ryegrass in a climate chamber: to test the method on a monocot plant that is not a cereal crop, ryegrass was grown in a climate chamber of the same type described above. Plants were cultivated in a commercial potting mix substrate (‘Spezialmischung 209’, RICOTER Erdaufbereitung AG, Aarberg, Switzerland) with a light/dark photoperiod of 16/8h. The average day/night temperature was 25/15°C, and relative humidity was kept at 50±15%. We tested five genotypes, each with four replicates, and measured leaf elongation for 4 days.

All investigated field plants were fertilized during the measurement period with 40kg N ha^−1^ (Landor Nitrate, 27% N +2.5% Mg; Landor, Birsfelden, Switzerland), 40kg P_2_O_5_ ha^−1^, and 80kg K_2_O ha^−1^ (Agroline Concentro, 13% N, 13% P_2_O_5_, 26% K_2_O; Agroline AG, Roggwil, Switzerland), and were watered when necessary to exclude any confounding effect on plant growth except temperature.

### Experimental set-up of the leaf elongation measurements

For each investigated plant of a given species or genotype, we chose one new emerging leaf at the youngest measurable developmental stage. We used a small hairpin (25mm length, Fig. 1B) to connect the leaf tip to a 150-cm-long thread (Dyneema^®^ Fiber, ø 0.16mm, tensibility 1%; Climax, Ockert GmbH, Puchheim, Germany). The thread was guided through a plastic reverse roller to the black aluminium measurement panel (100×60cm). On the panel, the thread was guided by another two ball-bearing-mounted reverse rollers (35mm precision miniature bearing; Sapporo Precision Inc., Sapporo, Japan). The first roller guided the thread into the vertical plane of the panel and the second roller tensed the thread with a 20g counterweight in the back of the panel (commercial weights used by anglers, Fig. 1A, C, D).

A white bead (Polyoxymethylene^®^, 6g, ø 20mm; Maagtechnic, Füllinsdorf, Switzerland) was connected to the twine, which was able to move upwards and downwards on the vertical plane of the panel (Fig. 1D), guided by an aluminium u-rail (internal dimension 19mm). The panel was tilted by 5° backwards to force the beads to move along the u-rails. With leaf elongation, the bead was drawn upwards by the 20g counterweight on the back of the panel, which was balanced by the 6g weight of the bead and 1g roller resistance, resulting in 0.13N of tensile force exerted on the extending leaf. This tensile force is sufficient to gently stretch a leaf into the vertical plane, avoiding confounding effects by rain and wind, and has shown to leave natural leaf elongation unaffected (Gallagher *et al.*, 1976; Walter *et al.*, 2002; Sadok *et al.*, 2007). We built four panels, each providing 23 u-rail measurement positions from which three were used as reference positions (not connected to leaves). The panels were attached to two iron rods (2 m, ø 2cm) that were inserted ~40cm into the soil (field) or that were clamped to the testing table (climate chamber). In the field, panels were stabilized by two additional iron bars/poles to minimize wind movements (Fig. 1D).

Digital images (grey scale) were collected with a tripod-mounted LUPUSNET HD camera [1920×1080 pixels (2.1 pixel mm^−1^); LUPUS-Electronics, Landau, Germany], which was positioned 2 m from the panel to monitor the white beads every 120s. Overnight, images were illuminated by near-infrared diodes in the camera. The zoom and focus of the camera were adjusted to fit the 100×60cm dimensions of the panel.

In the field experiments, we recorded temperature and weather data with a Hobo weather station (Onset Computer Corporation, Bourne, MA, USA). Air temperature (°C) was measured at 2 m and 5cm above soil level (‘plant height’), and soil temperature (°C) at 1cm and 10cm soil depth. Precipitation (mm), relative humidity (%), volumetric soil water content (Vol%), PAR (µmol m^−2^ s^−1^), and wind speed (m s^−1^) were also recorded. In the climate chamber experiment with barley, air temperature at plant height was measured by small temperature sensors (Onset Computer Corporation), and room temperature, relative humidity, and radiation (PAR) was logged by the climate chamber. In the climate chamber experiment with ryegrass, we measured meristem temperatures instead of air temperature at plant height with six type T needle thermocouples (ø 0.1mm; Omega, Stamford, CT, USA) and recorded data with a Campbell CR10X data logger (Campbell Scientific, Logan, UT, USA).

### Image processing

To extract LERs from the image sequences, we developed software in MATLAB 8.2 (The Mathworks, Natick, MA, USA) that we called ‘Leaf Length Tracker’ (LLT). The program can be operated by a simple graphical user interface (see detailed instructions in the software manual, Supplementary Fig. S1) and consists of three central parts: marker (bead) tracking, correction of lens distortion (rectification), and displacement determination.

To calculate the image positions of the beads, which we used as indirect artificial landmarks for leaf elongation, the marker tracking approach described in Mielewczik *et al.*, (2013) was applied. The algorithm is based on a cross-correlation algorithm with position interpolation for sub-pixel accuracy. After loading an image sequence into the tracking software, bead positions and surrounding search areas are set manually using the interface (Supplementary Fig. S1) and the position estimation is started for each bead, which is tracked throughout the image sequence of an experimental period (for a detailed description of the marker tracking algorithm, see Mielewczik *et al.*, 2013). To ensure high-quality template position (bead) tracking, a bead is considered to be lost when the cross-correlation coefficient (CC; quality of position localization) is below 0.5, which would result in unreliable results (typical values were 0.85–0.95, see ‘Results’). When losing a tracking position, the software tries to localize the bead in the next image of the sequence and deletes the displacement data of the particular bead in the problematic image (see Supplementary Fig. S1).

To compensate for lens distortion effects, we took a calibration image of a checkerboard (square size 45.5mm) of the size of the measurement panel before each experiment, positioned in the plane of the beads. The transformation parameters for rectification of the image were calculated and then applied to the pixel coordinates of the tracked beads, which resulted in bead positions in millimeters. By giving the size of the checkerboard squares, an accurate pixel size was determined for the rectified images and therefore for the corrected bead positions. To extract the displacement of the bead positions, a second order polynomial was fitted to all positions of each bead, onto which all positions were projected. Bead displacements were measured as distances along the polynomial projection in millimeters throughout the image sequence.

LLT can be downloaded at SourceForge (https://sourceforge.net/projects/leaf-length-tracker/) and is compiled for Microsoft Windows (64-bit). A user manual to operate the program is provided in Supplementary Fig. S1 at *JXB* online. LLT requires the MATLAB Compiler Runtime (MCR version R2013b; 8.2 64-bit; The Mathworks) to be installed on the user machine (download at http://www.mathworks.com/products/compiler/mcr/).

### Verification of image-based displacement measurements

**Fig. 2.**
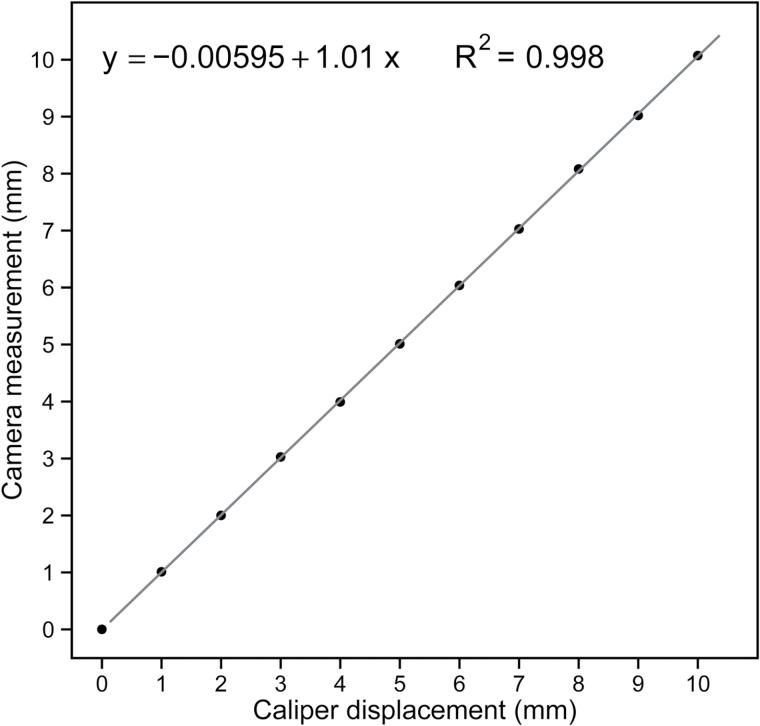
Linear correlation of image based displacement measurement against the manual calliper controlled movement of a bead. Similar correlations were found at each of the 23 panel positions.

We verified the accuracy of our measurement set-up at each of the 23 possible panel positions by manually moving the single measurement units (hairpin, twine, 20mm bead, and 20g counterweight) in steps of 10×1mm using a digital calliper (accuracy ±0.02mm; Series 500; Mitutoyo, Kawasaki, Japan). This was done under open-air conditions next to the Eschikon field site. We recorded the 1mm displacement steps of the beads with the camera and correlated the image-derived results against the manual measurements (Fig. 2).

### Leaf elongation calculation and statistics

LER were calculated per hourly intervals (mm h^−1^) as the ratios of leaf extension (ΔL, mm) per unit time (Δ*t*, h) using Eq. 1:

To correct LER for thread stretching by moisture and temperature or wind-induced movements of the panel, three reference measurements were taken throughout each measurement period by attaching the threads to a ground nail inserted into the soil (instead of a leaf). The mean displacement of the beads attached to those threads was subtracted from the measurements, which further improved the accuracy of the procedure (see Supplementary Fig. S2).

For graphics and statistics we averaged all temperature and weather data according to the LER intervals. We used simple linear regressions to describe the relationship between leaf growth and temperature. Here, air temperature at plant height (5cm above ground, in field experiments) and temperature in the meristem (ryegrass experiment in climate chamber) explained leaf elongation better (higher *R* ^2^-values, Supplementary Fig. S3) than air temperature 2 m above ground or soil temperature (5cm below ground).

For winter wheat, we corrected LER for thermal time using Eq. 2 as described in Reymond *et al.* (2003), but without considering vapour pressure deficit, to test the linearity of the genotype-specific temperature response:

where *a* is the linear coefficient between temperature (T, °C) and LER, with the intercept fitted through i. We set i = 0°C, because our studied species marginally (maximum ±0.26°C) deviated from this base temperature (T_b_ = 0°C, see ‘Results’).

For thermal time correction we calculated *a* (mm h^−1^ °C^−1^) using mean LER (fitted through zero) per variety and subtracted each observed single leaf LER.

To account for the genotypic response of LER to temperature we calculated *a* (mm h^−1^ °C^−1^) using Eq. 2 for each measured leaf in all experiments. To test for genotypic differences between *a*, a one-way ANOVA was conducted and genotypes were grouped by posterior testing using the Tukey-Kramer honest significant difference (HSD) test for each measurement period (week).

All growth rate calculations, statistical analyses, and diagrams were performed using R Statistical Software (version 3.0.2; R Development Core Team 2014) and the packages ‘ggplot2’ (Wickham, 2009) and ‘gdata’ (Warnes *et al.*, 2014).

## Results

### Method functionality and accuracy

Based on prior experience we found that white beads of 20mm diameter on a black background (100×60cm) were well suited for automated marker tracking throughout an image sequence. The high grey value contrast (white/black) enabled us to take measurements under all light conditions in the field, such as bright sunshine or fluctuating weather with transient shading by clouds. Owing to the solid construction of the aluminium panel, wind gusts up to 16 m s^−1^ did not affect our measurements (see Supplementary Fig. S4). Our four measurement panels functioned reliably and automated data collection for up to 7 days was possible in the growth chamber and in the field: even at the remote mountain study site in Kunkels (1180 m above sea level).

Application of LLT was straightforward and time efficient. The set-up of one measurement panel, the attachment of 20 leaves, and the installation of the camera took less than 1h. The subsequent software analysis of an image sequence of 5000 pictures required ~40min using a standard personal computer (Intel® Core™ i5 processor with 3.33 GHz CPU and 4.0 GB RAM). The CC of the bead position tracking was >0.95 in daytime images and >0.85 in night-time images. Bead position loss due to a poor CC <0.5 was observed rarely, mostly during bad weather conditions.

We tested the accuracy of our system by comparing manual 1.0mm stepwise displacement of the beads with image-based measurements and found the correlation between these was >0.998 (*R* ^2^) for each of the 23 bead positions (Fig. 2). When subtracting each of the image-based results by 1mm (1.0mm calliper controlled movements), the mean error of all 230 camera-derived measurements (10 measurements × 23 bead positions on the panel) was 0.029mm. We also checked the displacement of the fixed (non-moving) reference beads during these measurements and found they deviated on average 0.012mm, which we consider to be the technical resolution limit of our method.

### Leaf growth and temperature

**Fig. 3.**
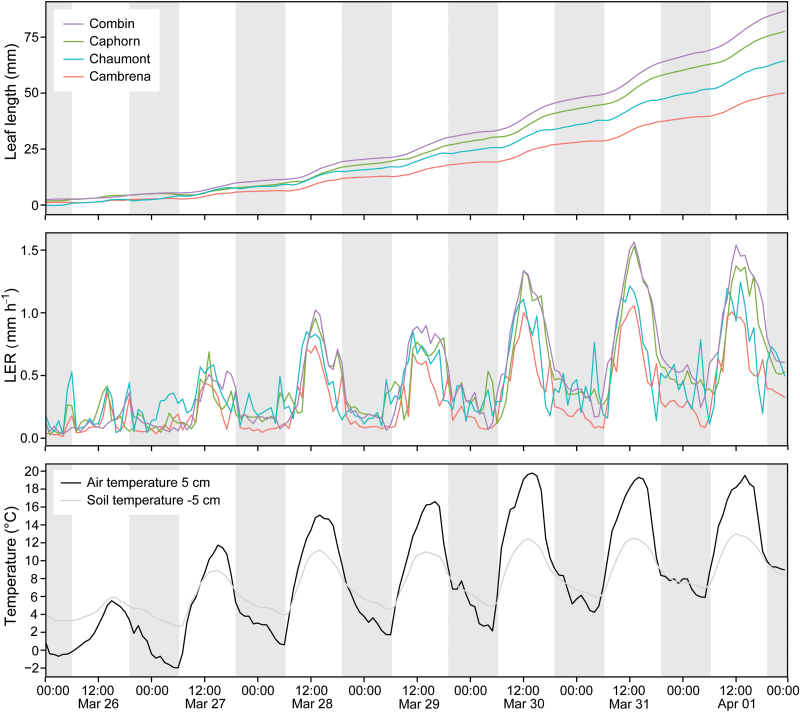
Time series of week 1 of leaf length measurements in four wheat varieties from 26th March to 2nd April 2014. Measurements were taken from leaf 7 when it first emerged until the leaves were fully developed. The upper graph is the mean cumulative leaf length of n = 20 leaves per wheat variety. The middle graph shows the corresponding mean LER. The lower graph shows the corresponding air temperature measured at 5cm above ground and the soil temperature measured 5cm below ground. Grey stripes indicate night hours.

We assessed the functionality of LLT by monitoring wheat and barley leaves at Eschikon and Kunkels in the field. The leaves of wheat and barley needed ~5–7 days to reach their final size under the prevailing low springtime temperatures in the field and in the barley climate chamber experiment. LERs precisely followed the daily temperature course (as shown for a representative period in Fig. 3). In winter wheat, LER was below 0.25mm h^−1^ with a few peaks up to 0.5mm h^−1^ at temperatures below 5°C, and consequently grew less than 5mm within the first 3 days. As soon as the air temperature, at 5cm height, rose above ~5°C, LER increased abruptly above 0.5mm h^−1^. With more pronounced diurnal temperature fluctuations, we found genotype-specific responses to temperature that became greater the higher the temperature rose (last 3 days in Fig. 3). Variety ‘Cambrena’ was most sensitive to low temperatures in week 1 and had the lowest growth rates during day and night. Following the morning increases in temperature, ‘Chaumont’ and ‘Caphorn’ showed earlier increases of LER. Once the daily temperature maximum was reached, ‘Combin’ and ‘Caphorn’ retained a high LER for longer than ‘Chaumont’ and ‘Cambrena’ (Fig. 3).

**Fig. 4.**
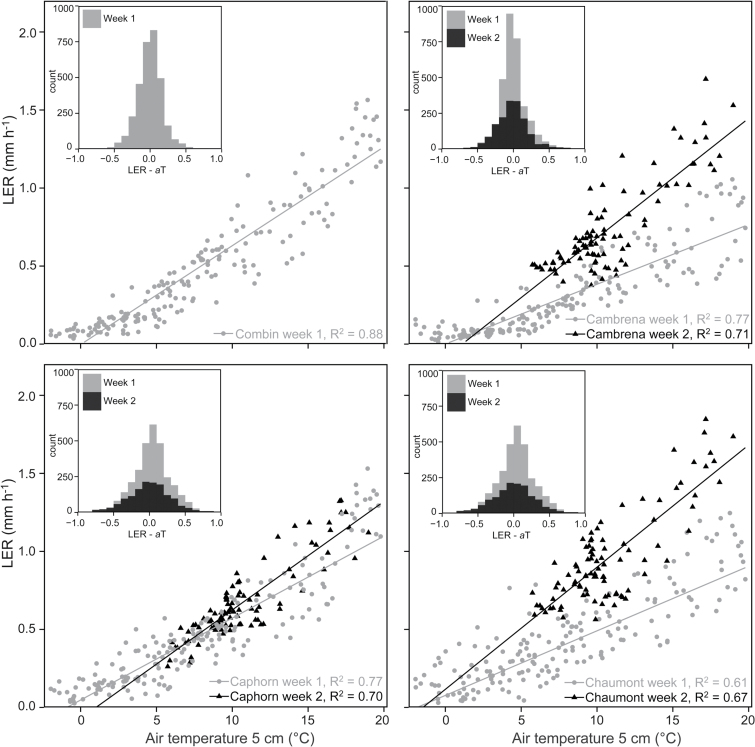
Linear correlations of mean LER (each n = 20 leaves) and air temperature at 5cm above ground for each of the four wheat varieties. Grey dots refer to week 1 (leaf 7) and black triangles to week 2 (leaf 8) of the experiment. The histograms in the upper left corners show LER corrected for thermal time (LER − *a*T) for week 1 and 2. Note: there is no data for week 2 for variety ‘Combin’.

Simple linear regressions of mean LER per wheat variety and air temperature resulted in *R* ^2^ > 0.61 (Fig. 4). The response of LER to temperature was different in week 1 and 2 depending on the temperature range the leaves were exposed to. ‘Combin’ was the most vigorously growing variety in week 1, when temperatures ranged from −2 to 20°C, whereas ‘Cambrena’ showed the least vigorous growth at these lower temperatures (Fig. 3 and 4). In week 2, when temperatures were always above 5°C, ‘Cambrena’ and particularly ‘Chaumont’ had a higher LER whereas ‘Caphorn’ did not profit from higher temperatures. The four wheat varieties also showed differences when correcting LER for thermal time (Fig. 4, LER − *a*T). The thermal time model showed a good fit for ‘Combin’ and ‘Cambrena’ in week 1, which indicates a linear temperature response (high peak in histograms of Fig. 4). However, the model underestimated *a*, the linear growth per mm h^−1^ °C^−1^, of the other two varieties in week 1 at temperatures >5°C and of all three varieties in week 2 at temperatures <15°C, which is also indicated by the small shifts to the left or right from the normal distribution in the histograms (Fig. 4).

When testing the *a* of single leaf linear correlations, we found the genotypic temperature responses shown in Fig. 4 to be significant for weeks 1 and 2 (ANOVA, *P* < 0.001, Fig. S4), which did result in significantly higher or lower *a* between genotypes in either measurement period (different letter in Fig. S5, Tukey-Kramer HSD test).

**Fig. 5.**
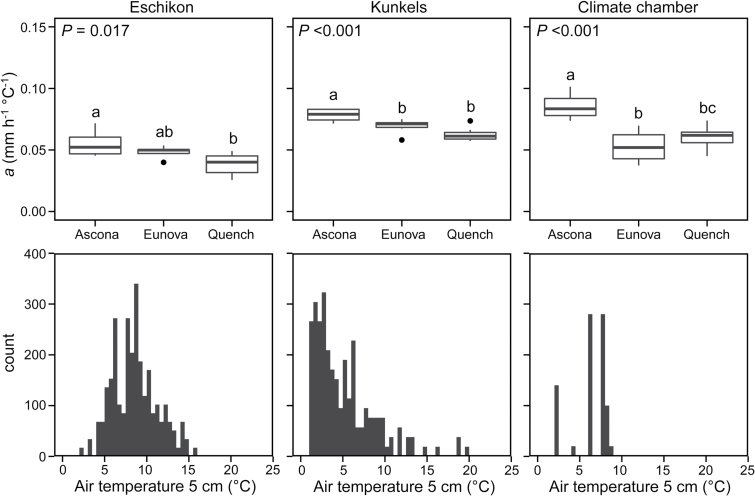
Upper row: LER per °C (*a*) of three summer barley varieties (each n = 7 leaves) from the two field sites and the climate chamber. *P*-values are derived from ANOVA and letters above boxes indicate significant genotype-specific differences (Tukey-Kramer HSD, *P* < 0.05). Lower row: Histograms of mean temperature per hour (in steps of 0.5°C) from the period of measurement.

We also identified a genotype-specific growth response to temperature in summer barley grown in the field as well as in the climate chamber (ANOVA, *P* < 0.01, Fig. 5). The variety ‘Ascona’ always showed a significantly higher *a* than the other varieties, independent of temperature range or field location, as well as in the very low temperature range (2–8°C) in the climate chamber. ‘Quench’ and ‘Eunova’ grew more slowly (lower *a*) in both field sites and the climate chamber, and were comparable with each other. ‘Quench’ showed significantly higher growth rates than ‘Eunova’ at the low temperatures in the climate chamber, and displayed a trend for lower growth rates at the higher temperatures in the two contrasting field sites, although these differences were not significant (Fig. 5, Tukey-Kramer HSD test). Thus, significant genotype-specific responses in barley were found when the temperature was very low or when the temperature range was large (~14K, Fig. 5).

In ryegrass, we found a trend for a genotype-specific reaction of LER to temperature in the five varieties, which were grown in a higher temperature range (15–25°C, Fig. S6). However, this was not significant when assessed by ANOVA (*P* = 0.29). Linear correlations using meristem temperatures resulted in higher *R* ^2^ values compared to correlations using the room temperature of the climate chamber. Furthermore, the room temperature of the climate chamber was slightly higher than meristem temperatures (see Supplementary Fig. S3).

The x-intercepts of LER plotted against temperature were close to 0°C for all studied species and periods when applying normal linear correlations without fitting the intercept through 0 (as shown for wheat in Fig. 4). There was no obvious trend among species (wheat: 0.02±0.15°C; barley: −0.15±0.26°C; ryegrass 0.18±0.22°C) or among genotypes in the specific periods (see Supplementary Figs S5, S6, and S7).

## Discussion

LLT has proven to be a precise method to study LERs of monocot plants and is particularly suited for field experiments. The measurement panel and image acquisition worked reliably in all four experiments. The software analysis ran robustly and automatically once an image sequence was loaded and the bead positions and search area were set. Software problems in prior attempts, such as a loss of the tracking regions during changing weather conditions, were overcome by the colour contrast (white beads/black background), and bead tracking resulted in high CC values (>0.85), indicating reliable measurements.

Leaf elongation instantly caused the movement of beads because of the low rolling resistance of the three reverse rollers. The tensile force exerted on the leaf added up to 0.13N, corresponding to a virtual, attached weight of 13g. The aluminium u-rails prevented any shaking of the beads and allowed upward or downward movements only.

The sensitivity of our method allowed us to record very low LERs at temperatures below 5°C of ~0.5mm h^−1^_,_ as well as high LERs of up to 1.5mm h^−1^ when temperatures rose during the day (Fig. 3).

We determined a measurement error of 0.03mm, which is small considering the error of the calliper (0.02mm) that we used to move the beads in steps of 1.0mm. We therefore interpret this error carefully given that we can not tell whether errors <0.02mm were due to the calliper precision limit or, more probably, caused by operator error during bead movement. However, when evaluating the non-moving reference beads during verification measurements, we arrived at a mean displacement of only 0.012mm. The software algorithm calculates the displacement from the previous position, thus any resulting error can be considered to be the true image-based resolution limit because these beads did not move. Yet, even a limit of 0.03mm allowed us to distinguish whether a leaf had grown in a 2min time interval at temperatures >5°C, during which their growth exceeded this ‘resolution limit’ by at least a factor of two. LERs at low temperature (<5°C) were very small but were still positive (first 2 days in Fig. 3); this would not have been the case had we recorded only error noise of ±0.03mm.

LERs below 0.25mm h^−1^ resulted in only minor leaf length increases and thus biomass accumulation would be negligible (first 3 days of Fig. 4). We cannot explore whether those small movements are elastic, reversible elongations due to a transient fluctuation in turgor or whether they are connected to underlying permanent cell elongation.

For all three species—winter wheat, summer barley, and ryegrass—we found T_b_ for leaf elongation close to 0°C with minor differences between species and genotypes (see Supplementary Figs S5, S6, and S7). These thermal limits are in accordance with the literature for wheat and barley, which are reported to be between 0 and 1°C (Gallagher and Biscoe, 1979; Gallagher *et al.*, 1979; for a review see Porter and Gawith, 1999) but lower than those for ryegrass, which has a reported T_b_ of ~4 °C (Peacock, 1975a).

Our growth rates for wheat, barley, and ryegrass (between 0.5 and 2.5mm h^−1^) are similar to LERs that have been obtained using other methods, such as linear variable displacement transducer or manual ruler measurements (Peacock, 1975a; Gallagher *et al.*, 1976), under comparable growth conditions in a temperature range of 5 to 25°C. However, LLT enables highly temporally resolved and precise data collection over periods of 7 days without interruption. This allowed us to observe LERs close to the thermal limit of growth (T_b_). We were able to detect short-term and genotype-specific reactions of LER to temperature, which were found to be significant in wheat and barley in the field and the climate chamber (Figs 4 and 5).

The temperature regime the leaves were exposed to had a large influence on the growth performance of the specific genotypes. Two wheat varieties, ‘Cambrena’ and ‘Chaumont’, had a higher *a* at temperatures above 5°C in week 2. ‘Caphorn’ did not profit from the higher temperatures in week 2, but grew better in week 1 when temperatures were mostly below 10°C (Fig. 4). Similarly, the barley variety ‘Eunova’ grew faster at higher temperatures, whereas the LER of ‘Quench’ did not increase as much as that of ‘Eunova’ or ‘Ascona’, the latter of which had the highest LER of the three varieties independent of the temperature range. These specific reactions to temperature underline the potential of different genotypic material regarding growth.

Applying linear correlations to explain growth in relation to temperature worked well in the temperature range of 5–15°C. However, our data suggest a non-linear relationship at temperatures below 5°C (wheat varieties ‘Combin’ and ‘Cambrena’; Fig. 4) and above 15°C, where LER was higher than predicted by the linear model, with the majority of data points situated above the regression line in both cases (Fig. 4). Small shifts of the peak from a normal distribution (LER – *a*T, histograms in Fig. 4) support a non-linear influence. Deviations from the thermal time model are again species specific, given that varieties ‘Caphorn’ and ‘Chaumont’ still showed a linear response at low temperature.

LLT can be used to record LER data with a single recording unit (camera, panel, and beads), which produced statistically robust results when measuring 20 leaves per crop variety (winter wheat data) and allowed fast screening of genotypes when measuring 6×3 leaves per variety (barley experiment) or 4×5 leaves (ryegrass experiment). The ability to simultaneously measure hundreds of plants is easily within reach: the described set-up is inexpensive, thereby opening up the possibility of applying multiple measurement panels or even double the number of measurement position per unit. Particularly, monitoring growth throughout the entire development course, *in situ*, of the studied leaves to our knowledge has not yet been achieved under field conditions.

Our method pushes forward the state of the art in plant growth measurements and allows corroboration between a controlled laboratory environment and field conditions, which is a recognized gap in the technical ability of researchers (Dhondt *et al.*, 2013; Araus and Cairns, 2014; Nelissen *et al.*, 2014; Wuyts *et al.*, 2015). Testing plant–environment interactions with regard to molecular traits or rising climatic stress factors such as temperature or drought is an important tool for future plant breeding (Araus and Cairns, 2014; Grieder *et al.*, 2015) and will contribute to the basic understanding of plant physiology. Our method might also be useful for studying other graminoids and many other treatment factors.

## Supplementary data

Supplementary material are available at *JXB* online. Additional file contains Supplementary Fig. S1. Additional file two contains Supplementary Figs S2–S7.

Figure S1. A step-by-step manual guiding users through the software analysis of an image sequences.

Figure S2. The result of reference bead subtraction.

Figure S3. Linear correlations of LER to different temperature measurements.

Figure S4. Linear correlation of LER and wind gust speed.

Figure S5. Boxplots of significant genotype-specific leaf growth per °C in winter wheat, x-intercepts, and temperature histograms.

Figure S6. Boxplots of ryegrass leaf growth per °C, x-intercepts, and temperature histograms.

Figure S7. X-intercepts of summer barley from linear correlations of LER and temperature.

Supplementary Data
