# Supplementary material for: Leaf Length Tracker: a novel approach to analyse leaf elongation close to the thermal limit of growth in the field
Source: J Exp Bot. 2016 Jan 27;67(6):1897–906. doi: 10.1093/jxb/erw003 (PMC4783369; doi:10.1093/jxb/erw003)
Supplement: Supplementary Data [file supp_67_6_1897__index.html]

Leaf Length Tracker: a novel approach to analyse leaf elongation close to the thermal limit of growth in the field — Leaf Length Tracker: a novel approach to analyse leaf elongation close to the thermal limit of growth in the field — Supplementary Data 

# Leaf Length Tracker: a novel approach to analyse leaf elongation close to the thermal limit of growth in the field

## Supplementary Data

Data files

- Supplementary\_figure\_S1.pdf - Supplementary Data
- Supplementary\_figure\_S2\_figure\_S7.pdf - Supplementary Data
