# Supplementary material for: Leaf Length Tracker: a novel approach to analyse leaf elongation close to the thermal limit of growth in the field
Source: J Exp Bot. 2016 Jan 27;67(6):1897–906. doi: 10.1093/jxb/erw003 (PMC4783369; doi:10.1093/jxb/erw003)
Supplement: Supplementary Data [file supp_erw003_Supplementary_figure_S1.pdf]

Leaf Length Tracker: A novel, highly sensitive method for field use to analyze leaf elongation in monocot plants

S Nagelmüller[1 2], N Kirchgessner[1], S Yates[1], M Hiltpold[-1], A Walter[1]

[1] Institute of Agricultural Sciences, Swiss Federal Institute of Technology, Universitätstrasse 2, 8092 Zurich, Switzerland

[2] Institute of Botany, Department of Environmental Sciences, University of Basel, Schönbeinstrasse 6, 4056 Basel, Switzerland

Figure S1: Manual for Leaf Length Tracker (LLT) version 1.01

Software written by Norbert Kirchgessner (Group of Crop Science, Institute of Agricultural Science, ETH Zurich, Switzerland, contact: cropscience_soft@usys.ethz.ch)

Introduction:

The software is made for tracking artificial landmarks (beads) and calculating leaf elongation rates using an image sequence of the leaf length measurement panel. It is free for download at https://sourceforge.net/projects/leaf-length-tracker/.

It is written in Matlab and compiled for standalone usage in Version 2013b 64 Bit on Windows. For running it, MS Excel and the Matlab MCR 2013b (available for free at http://ch.mathworks.com/products/compiler/mcr/) must be installed on the computer. We recommend to use a computer with enough RAM to process the data (we used an Intel® CoreTM i5 processor and 4 GB RAM).

The provided test data at Sourceforge is a short and reduced image sequence compared to the original data used in the article and therefore only useful for a fast general check of the principles of the software and the requested file structure.

Requirements:

An image sequence of your measurement panel with a time interval of your choice and a calibration image of a checkerboard (we used a square size of 45.5 mm) of the size of your panel. Markers must move in y-direction.

Each image in the sequence needs to be named in the following format:

20141119_CH01_164414s.jpg

where the first number is the date information (yyyy,mm,dd). The second term CH01 is a name code for your image sequence (choose CH01-CH99). And the last number the time information (hh,mm,ss). The recommended image resolution is at least 2 pixel mm^{-1}.

If you are using a LupusNET HD camera (LUPUS-Electronics, Landau, Germany), you can use the program “Invid_lupus_read” also contained in the zip-file to rename your images. You can also use the program “Bulk Rename” (http://www.bulkrenameutility.co.uk/Download.php) to name your images accordingly.

All images need to be in the same folder, which should also contain another subfolder named “Calibration”, containing the checkerboard calibration image.

Now you are ready to start the analysis following the instructions.

1. Open a Dos window by opening start menue, searching for “cmd” and executing cmd.exe. Drag and drop Leaf_lenght_tracker.exe into the shell window, activate it by mouse click, and press “enter”.

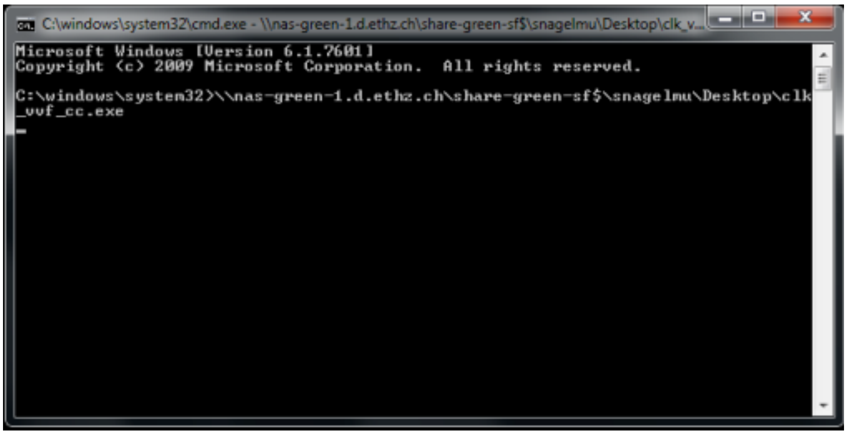

2. If you are using “Leaf Length Tracker” for the first time, the following license agreement opens. Klick “I read the complete above license agreement. I understand and agree” to proceed.

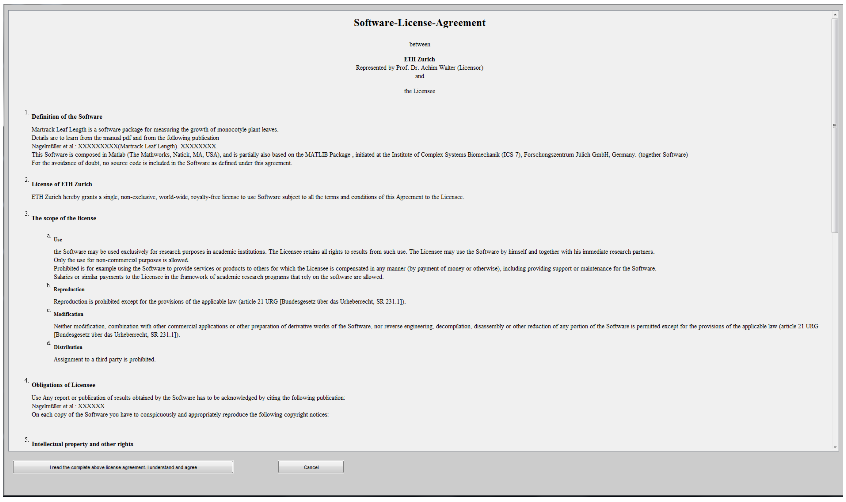

3. The software interface opens up and provides the single analysis steps in the recommended order: Track mackers, Rectification (Correction for lens distortion) and calculation of displacements. You can also choose the programms individually once the previous analysis has been done before. Choose “Track makers” to start the tracking procedure.

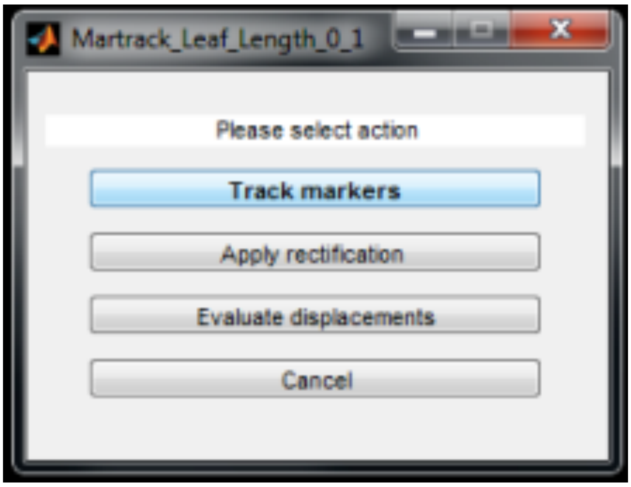

4. A new window opens and asks, if you want to save each image with the tracked regions. Those images show the tracked bead position and are saved as a TIFF stack. (If you chose yes, the evaluation will take noticeably longer.)

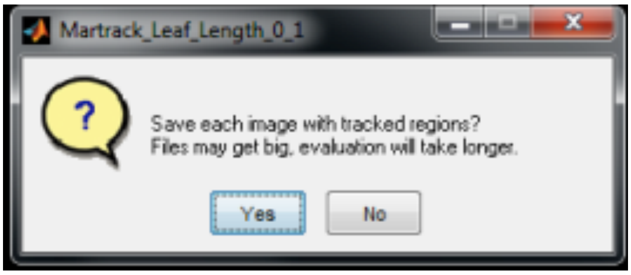

5. The next window asks if you want the programm to contiue the analysis if a region (bead) is lost. If answering “yes”, the software tries to localize the bead in the next image of the sequence and deletes the displacement data of the particular bead in the problematic image. When clicking “no”, the program stops the tracking of the particular bead.

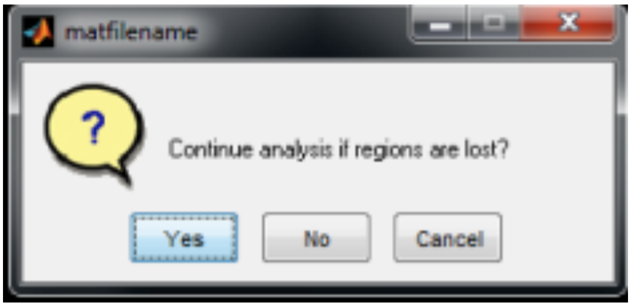

6. Browse to your image folder and select the first image of your sequence

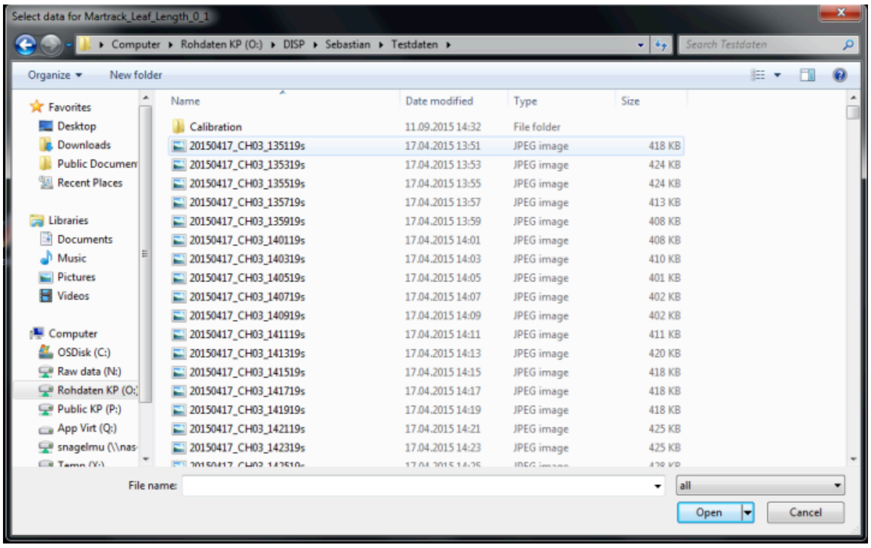

7. The program opens the first image were the markers need to be selected by mous-klicking into the center of the beads. Backspaces remove the last marked position. Double-clicks or „enter“ finish the bead selection. We recommend to select the three reference beads first (lower three beads in image below).

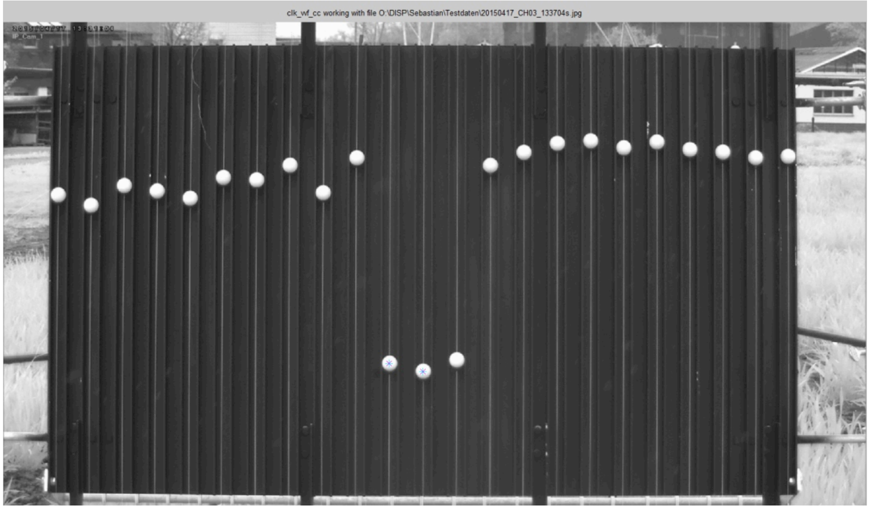

8. Now you are asked to set the template size around the beads. Typ a new template size, and click „Try“ to visualize as image overlay. As soon as the template size fits well arround the beads, click “Ok” to continue.

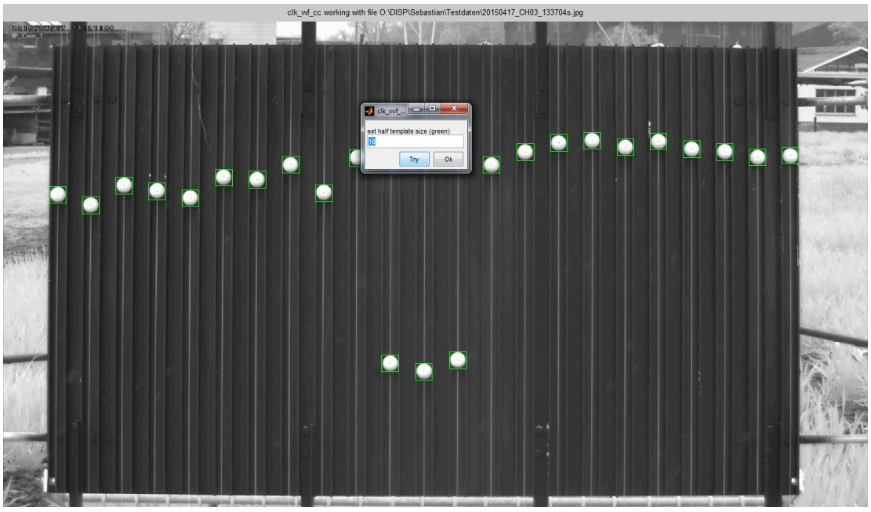

9. As a last step you have to define the search length around the beads. Type a new search length, and visualize the corresponding search area as image overlay by clicking “Try”. We recommend a seach area size between 30 and 40 pixel. Click “Ok” to continue, which starts the tracking.

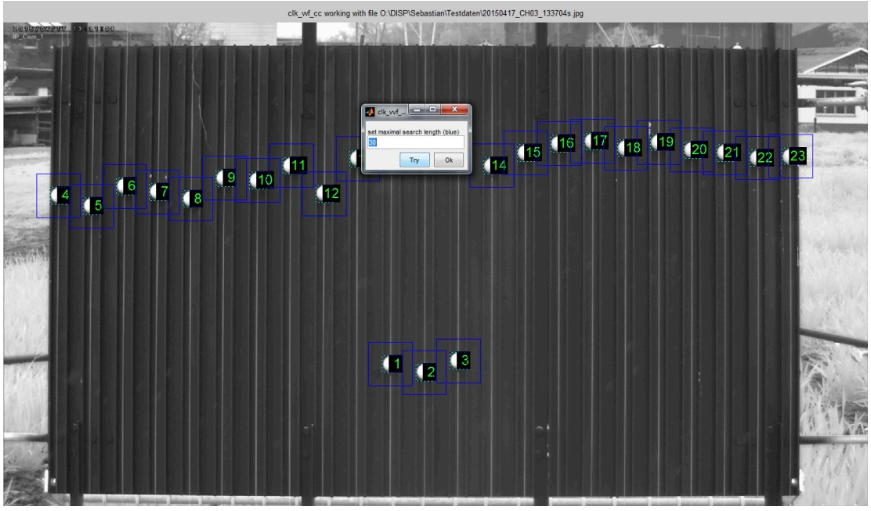

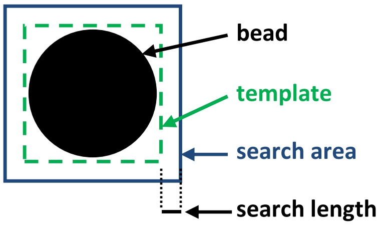

© Mielewczik et al. (2013)

10. Now the calculation starts, and every bead is tracked throughout the whole sequence. The following window opens when the tracking is finished.

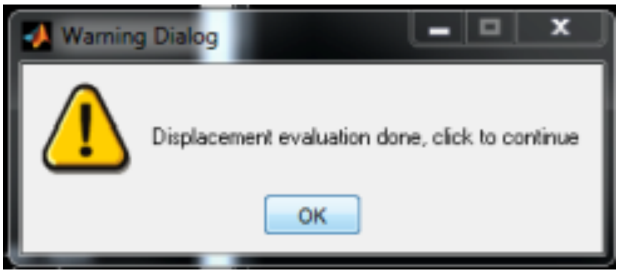

After clicking “Ok” figures with the crosscorrelation coefficient of regions over the image sequence and the number of found regions over the frame number are displayed. The tracking dispalcements are stored in the result subfolder “VVF_eth”, which will be used for calculating leaf length displacements in milimeters.

11. The second step in the menue is the “Apply rectification” function, wich corrects your images for lens distortion and automtically converts the pixel coordinates into milimeters.

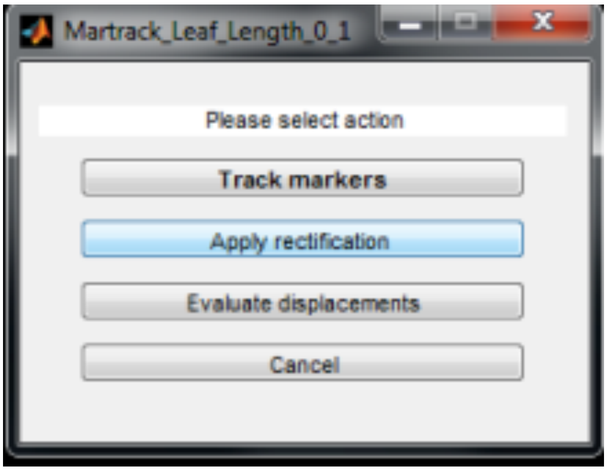

12. Browse to your folder containing the “Calibration” folder and choose “Select Folder”.

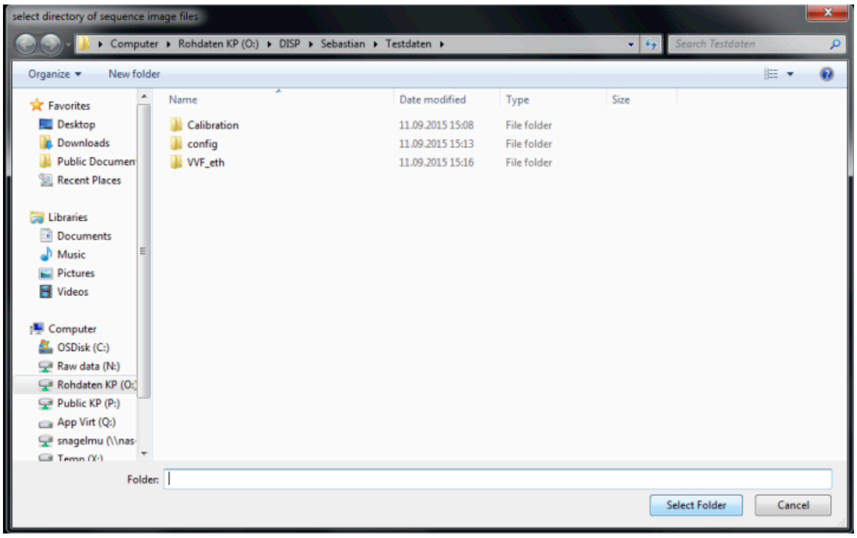

13. A window opens and ask for the size of the calibration squares. Typ the square size in milimeters and click “OK”.

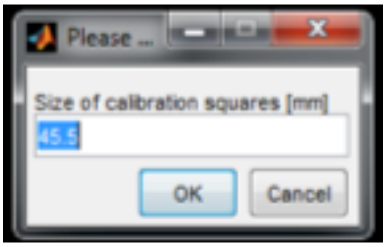

14. Images of the checkerbord and the first image in the sequence are displayed in the “original” and “corrected” version and stored in the subfolder “Check” in the “Calibration” folder.

15. To finish the analysis, select “Evaluate displacements” in the user interface.

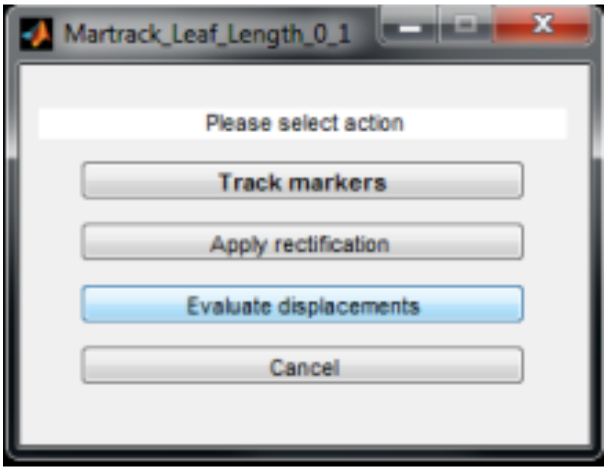

16. Again, browse to your image folder and mouse-clike on the folder “VVF-eth” and click select folder.

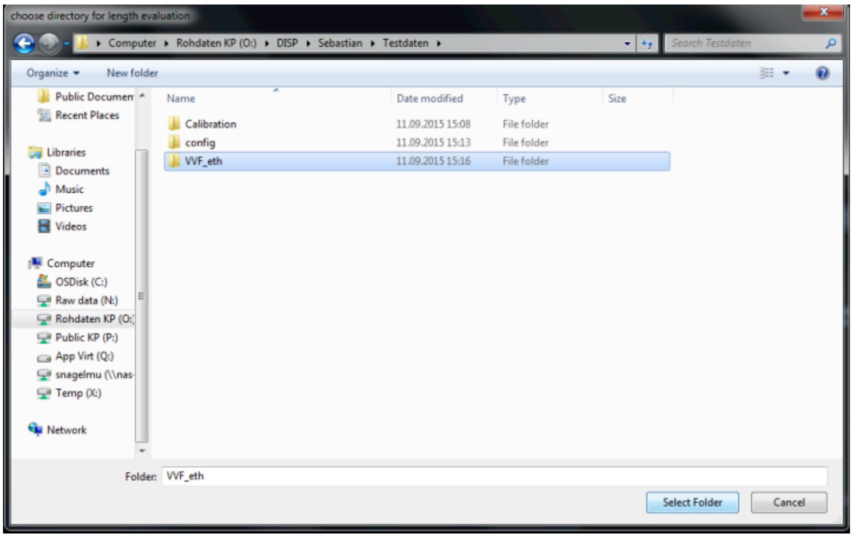

17. A window informs you that data, corrected for lense distortion is used. You can close it by clicking ok.

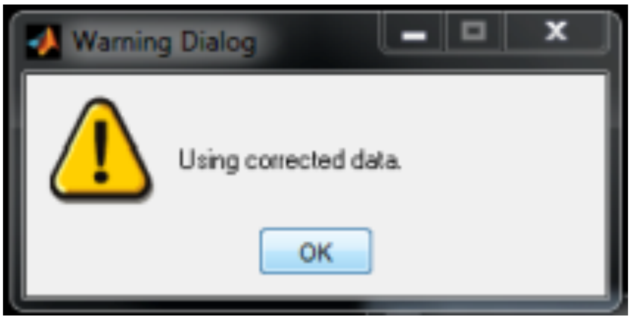

18. Exit the software by clicking “Cancel” in the user interface

When calculations are finished figures with displacement (mm) and LER (mm h^{-1}) over time are displayed. In the subfolder "Length_sph1_corrected" in the result folder "VVF_eth" you can find different figures and a Microsoft excel-file containing an extra sheet for each tracked bead Lx – Ly (with columns "displ (mm)" for displacement e.g. leaf length, "displ (mm) smoothed", "d displ smoothed / d time (mm/h)" e.g. leaf elongation rate and the "date" in the format dd.mm.yyyy hh:mm).

Now you can start data analysis and work with the leaf length data (displ) or elongation rates (d displ smoothed / d time (mm/h)). The elongation rates presented in the article were calculated with the R Statistical Software (R Core Team 2014) using the "displ (mm)" and "date" columns. We therefore recommend R for data analysis and the package "gdata" (Warnes et al. 2014) to read in the result excel-file.

Warranty

The Software is provided "as is". ETH Zurich does not make any warranty of any kind. Disclaimed warranties include for example:

Liability

ETH Zurich disclaims all liabilities. ETH Zurich shall not have any liability for any direct or indirect damage except for the provisions of the applicable law (article 100 OR [Schweizerisches Obligationenrecht]).
