# Supplementary material for: Leaf Length Tracker: a novel approach to analyse leaf elongation close to the thermal limit of growth in the field
Source: J Exp Bot. 2016 Jan 27;67(6):1897–906. doi: 10.1093/jxb/erw003 (PMC4783369; doi:10.1093/jxb/erw003)
Supplement: Supplementary Data [file supp_erw003_Supplementary_figure_S2_figure_S7.pdf]

Leaf Length Tracker: A novel, highly sensitive method for field use to analyze leaf elongation in monocot plants

S Nagelmüller[1 2], N Kirchgessner[1], S Yates[1], M Hiltpold[-1], A Walter[1]

[1] Institute of Agricultural Sciences, Swiss Federal Institute of Technology, Universitätstrasse 2, 8092 Zurich, Switzerland

[2] Institute of Botany, Department of Environmental Sciences, University of Basel, Schönbeinstrasse 6, 4056 Basel, Switzerland

Figure S2:

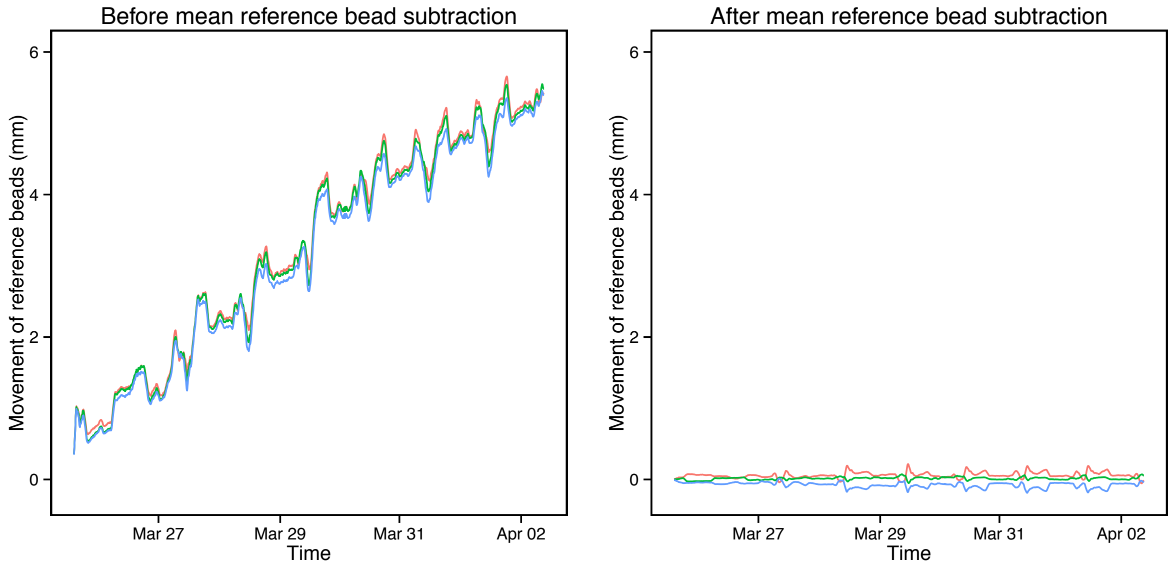

Left: Example for the movement of three reference beads over time (taken from the wheat measurement in week 1) that were not attached to leaves. Short-term movements are due to wind and moisture induced thread stretching, the long-term displacement can be explained by small position shifts of the camera tripod relative to the measurement panel. Right: The result when subtracting the mean reference bead displacement from reference beads itself, which improved the accuracy of our measurments.

Figure S3:

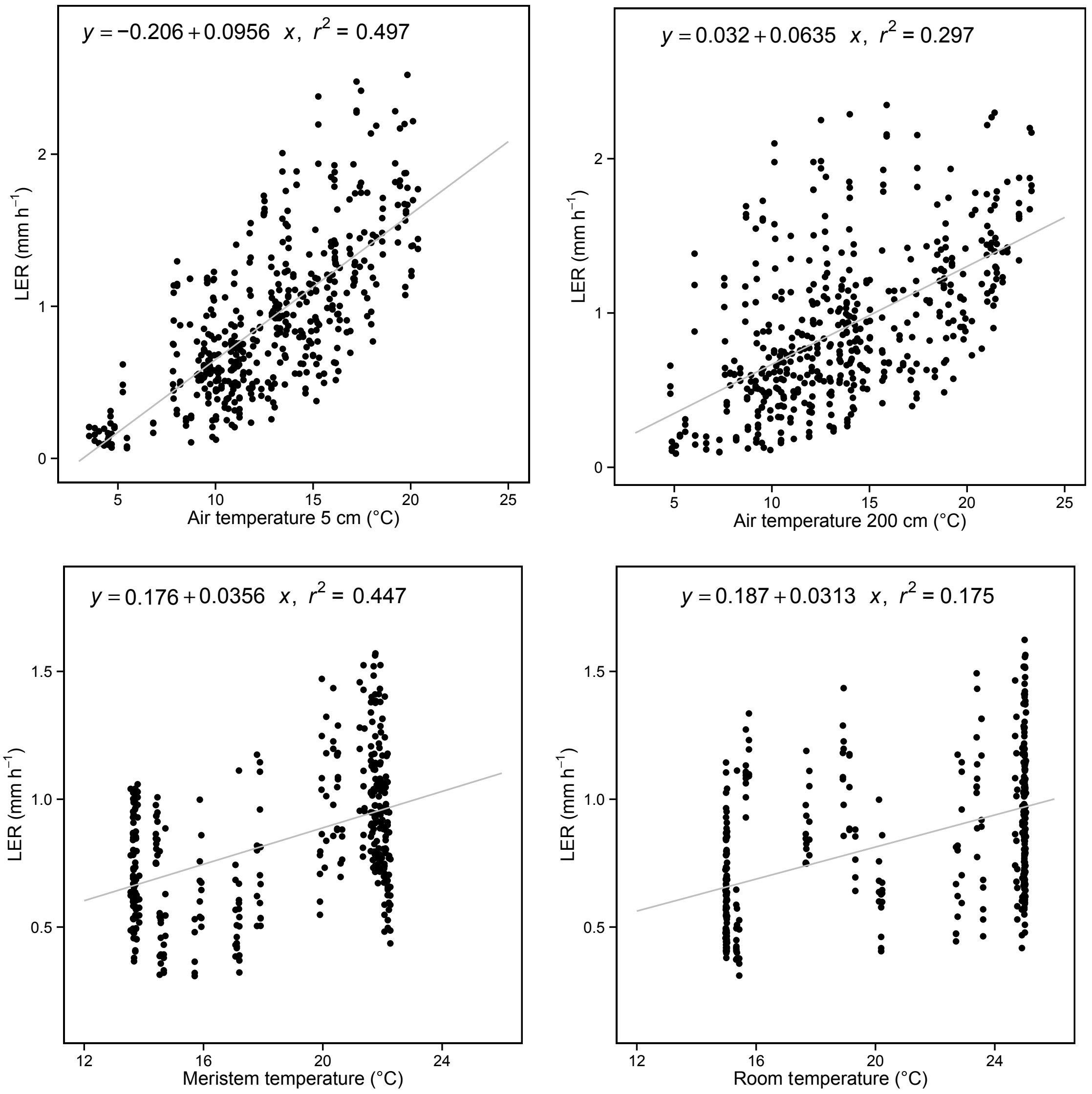

Upper row: Correlation of mean LER of summer barley (example from the Kunkels study site) to temperature at plant height (air temperature 5 cm) and in 200 cm height. Note the higher R^2 values for temperature data in 5 cm and the slightly different temperature range. This was consisted in all field measurements. Lower Row: Correlation of mean LER of ryegrass using meristem temperature (left) and room temperature of the climate chamber. Again, note the higher R^2 values for meristem temperature data and the slightly different temperature range.

Figure S4:

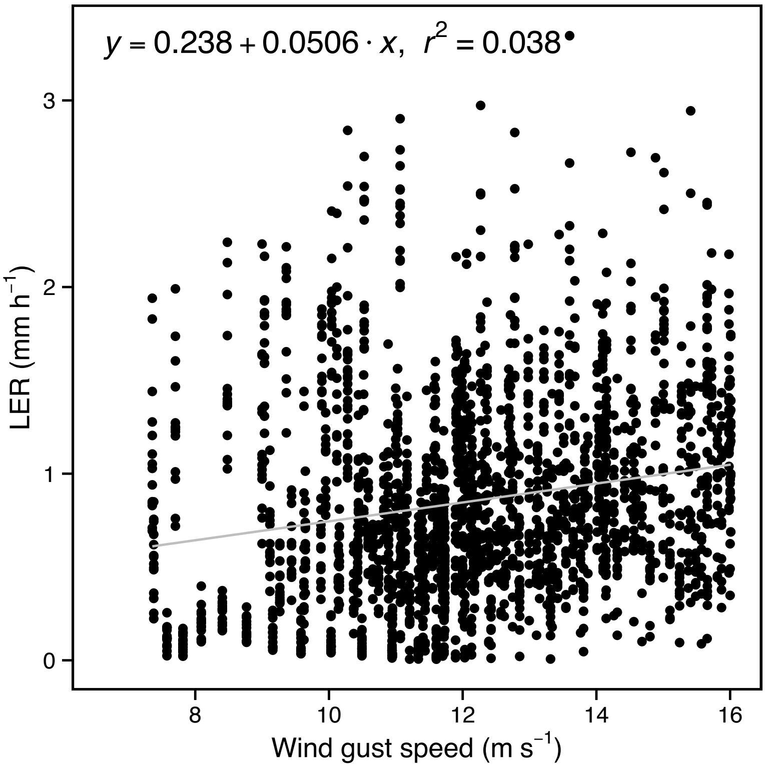

Correlation of LER of summer barley against wind gust speed from the most windy weather period at the mountain Kunkels study site. There is no correlation with wind gusts of up to 16 m s^{-1}.

Figure S5:

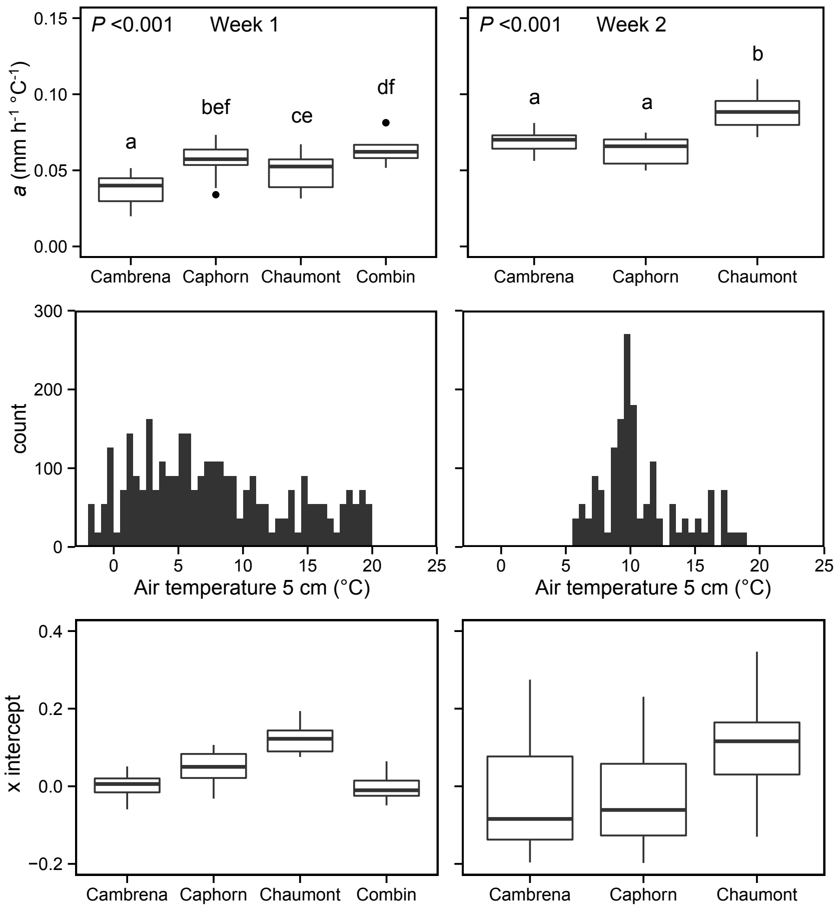

Upper row: LER per °C (*a*) of four winter wheat varieties (each n = 20 leaves) from week 1 (left) and week 2 (right). *P*-values are derived from ANOVA and letters above boxes indicate significant genotype-specific differences (Tukey-HSD, P <0.05). Middle row: Histograms of mean temperature per hour (in steps of 0.5 °K) from the period of measurements. Lower row: X intercepts close to 0 °C for all wheat genotypes (derived from normal linear correlations of LER and temperature without fitting trough zero). This did slightly change the ANOVA *P*-values but did not change the significance or Tukey-HSD results.

Figure S6:

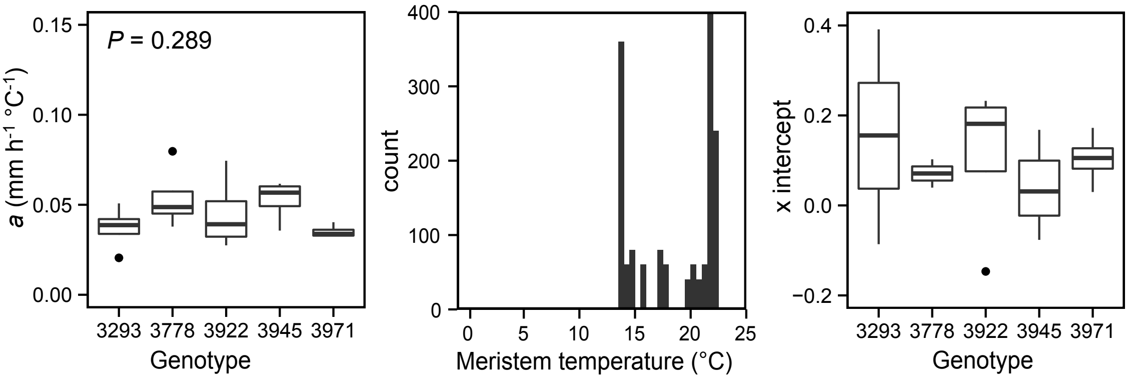

Left: LER per °C (a) of five ryegrass genotypes (each n = 4 leaves). *P*-value is derived from ANOVA. Middle: Histograms of meristem temperature per hour (in steps of 0.5 °K) from the period of measurements. Right: X intercepts close to 0 °C for all genotypes (derived from normal linear correlations of LER and temperature without fitting trough zero).

Figure S7

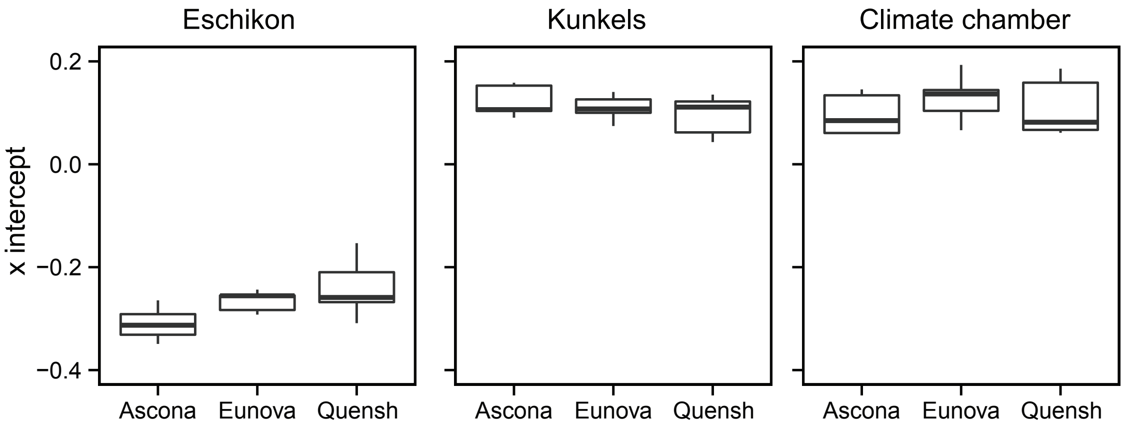

X intercepts close to 0 °C for all three summer barley genotypes at the two field sites and in the climate chamber (derived from normal linear correlations of LER and temperature without fitting trough zero). Again, this did slightly change the ANOVA P-values presented in the article but did not change the significance or Tukey-HSD results.
